# The Role of the RNA-Binding Protein Family MEX-3 in Tumorigenesis

**DOI:** 10.3390/ijms21155209

**Published:** 2020-07-23

**Authors:** Simon Jasinski-Bergner, André Steven, Barbara Seliger

**Affiliations:** Institute for Medical Immunology, Martin Luther University Halle-Wittenberg, 06112 Halle (Saale), Germany; simon.jasinski@uk-halle.de (S.J.-B.); andre.steven@uk-halle.de (A.S.)

**Keywords:** MEX-3, immune evasion, HLA class I, antigen presentation, antigen processing, cancer

## Abstract

The muscle excess 3 (MEX-3) protein was first identified in *Caenorhabditis elegans (C. elegans)*, and its respective homologues were also observed in vertebrates, including humans. It is a RNA-binding protein (RBP) with an additional ubiquitin E3 ligase function, which further acts as a post-transcriptional repressor through unknown mechanisms. In humans, MEX-3 proteins post-transcriptionally regulate a number of biological processes, including tumor immunological relevant ones. These have been shown to be involved in various diseases, including tumor diseases of distinct origins. This review provides information on the expression and function of the human MEX-3 family in healthy tissues, as well after malignant transformation. Indeed, the MEX-3 expression was shown to be deregulated in several cancers and to affect tumor biological functions, including apoptosis regulation, antigen processing, and presentation, thereby, contributing to the immune evasion of tumor cells. Furthermore, current research suggests MEX-3 proteins as putative markers for prognosis and as novel targets for the anti-cancer treatment.

## 1. Introduction

RNA-binding proteins (RBPs) play a critical role in the regulation of various RNA processes. After binding to their target RNA molecules, they form ribonucleoprotein complexes to regulate important processes, including splicing, cleavage, and polyadenylation, transport, translation, and degradation of coding and even different non-coding RNA species, such as long non-coding RNAs (lnRNAs) and microRNAs (miRNAs), as well as their precursors. It is estimated that mammalian cells harbor >1000 RBPs [1].

In general, RBPs contain RNA-binding domains (RBDs) for the binding of a special RNA sequence motif or structural motifs like the RNA recognition motif, heterogeneous ribonucleoprotein (hnRNP) K homology domain (KH), or the DEAD box helicase domain [2,3,4,5]. Recently, studies identified a novel class of RBPs lacking canonical RBDs, but containing unorthodox binding sites for RNA interaction [1,2]. Despite many computational methods being reported to predict RBP binding sites by in silico sequence analyses [6], this new group of RBPs circumvents the possibility of in silico prediction for their binding sites in RNA molecules. Furthermore, functional analyses for accurate RBP target validation are still mandatory.

Concerning their activity, RBPs are involved in a broad spectrum of physiological and patho-physiological processes, like autoimmune diseases and cancer. Thus far, numerous RBPs have been described, which are involved in the initiation and progression of tumors [2,7]. These include, among others, the evolutionally conserved RBP family MEX-3 (muscle excess 3), consisting of four members (MEX-3A-D). All four MEX-3 proteins contain two RNA-binding KH domains and an ubiquitin E3 ligase RING domain [8], suggesting that E3 ligases not only affect the protein fate, but also the RNA decay, through mechanisms that are unknown, thus far [9]. Members of the human MEX-3 family are not only involved in homeostasis and balance between cell self-renewal and differentiation, but also in the processes of malignant transformation. MEX-3A and MEX-3C proteins were recently reported to act as negative post-translational regulators of several target genes. They mediate ubiquitination, leading to a proteasome-dependent degradation of their target proteins, also including cancer-relevant target proteins like the tumor suppressor RIG-I, which could be ubiquitinated by MEX-3A, during glioblastoma tumorigenesis [10]. Interestingly, the ubiquitination of the RIG-I protein was also reported for the MEX-3C protein [11]. Next to this, the MEX-3 proteins also exert an additional and so far not completely understood mechanism of target mRNA ubiquitination, which causes mRNA decay, affecting the post-transcriptional gene regulation of several target genes, including HLA class I genes [12]. Dependent on the tumor type and family member, *MEX*-*3* expression correlates with an increased or reduced patients’ survival. This review summarizes the expression and role of human MEX-3 proteins in tumorigenesis, as well as its potential as a putative target for anti-cancer therapy.

## 2. History of MEX-3

MEX-3 is a RNA-binding translational repressor first discovered in nematode *C. elegans*, with a role in the embryonic and post-embryonic development. It contains two tandem repeat KH domains characterized as a conserved region interacting with the 3′ untranslated region (UTR) of its target PAL-1 and inhibits its translation [13].

In 2006, Andrew Fire and Craig Mellow earned the Nobel Prize in Medicine and Physiology for the discovery of RNA interference, using e.g., RNA interference against MEX-3 mRNA through transfection of its anti-sense RNA into *C. elegans* embryos. In the case of a complete MEX-3 mRNA knockdown, an embryonic arrest was observed [14].

Mutations in the *MEX*-*3* gene disrupting its expression are lethal. MEX-3 is involved in cell fate specification in the early embryonic stage and contributes to the maintenance of the totipotency of the germ line in adult worms. In *C. elegans*, it represents a fundamental regulator of asymmetry, mediating the early steps of cell fate determination [13]. In adult *C. elegans*, MEX-3 is involved in the maintenance of germ stem cells [15]. Despite an extensive existing knowledge of MEX-3 in *C. elegans*, the function of MEX-3 family members in vertebrates are not well characterized.

## 3. Characteristic Features of Human MEX-3 Family Members

Human MEX-3 represents a family of evolutionarily highly conserved RBPs. The human MEX-3 proteins belong to the heterogeneous nuclear ribonucleoprotein (hnRNP) family of RBPs. They share a high homology with the *C. elegans* MEX-3. In human and murine genomes, four MEX-3 homologous proteins, named MEX-3A, -3B, -3C, and -3D, were identified [8], which share a high homology to MEX-3 of other vertebrates. These four murine MEX-3 proteins are approximately 95% identical at the amino acid level to the human counterparts [8]. The MEX-3A-D proteins consist of two KH RNA-binding domains at the N-terminus, a zinc finger (ZNF) domain, which mediates the protein interactions at the C-terminus and an ubiquitin E3 ligase RING domain, which is absent in the MEX-3 protein of *C. elegans* [8]. The human *MEX*-*3* genes are located on different chromosomes—*MEX*-*3A* is encoded on chromosome 1q22, *MEX*-*3B* on chromosome 15q25.2, *MEX*-*3C* on chromosome 18q21.2, and *MEX*-*3D* on chromosome 19p13.3. Interestingly, the chromosomal region 19p13.3 was classified as a fragile chromosomal region, frequently deleted in various human cancers [16]. In colorectal cancer, *MEX-3C* was identified as a cancer chromosome instability gene, which is frequently lost in CIN^+^ tumors [17].

**Figure 1:**
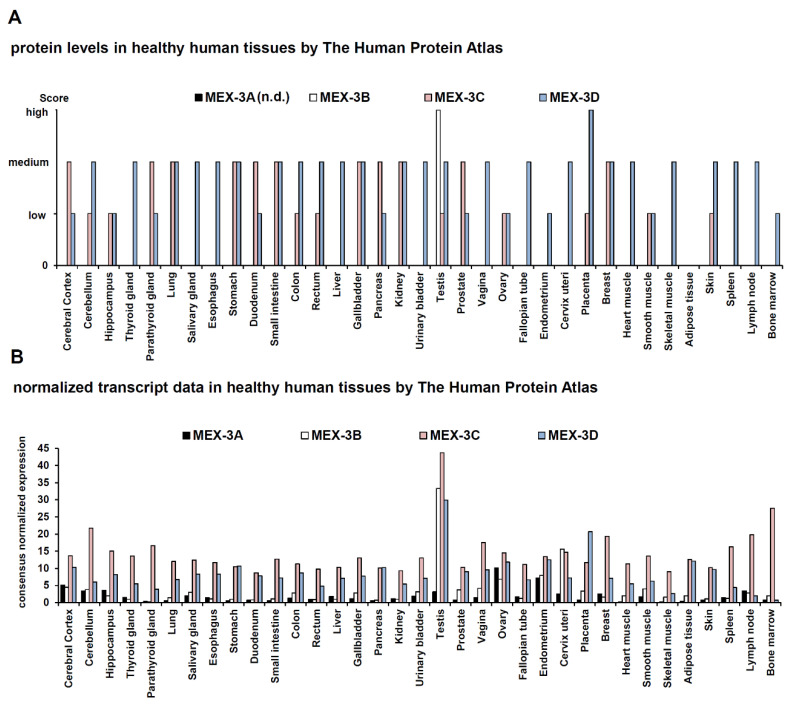
Protein and mRNA levels of MEX-3A-D in healthy human tissues of distinct origin. The protein (**A**) and mRNA (**B**) levels of the MEX-3A-D in selected healthy human tissues are expressed as bar diagrams based upon the data sets of The Human Protein Atlas [20,21,22]. Protein data for MEX-3A were not available. The protein levels were scored using immunohistochemistry data and the mRNA data are expressed as consensus normalized expression levels created by combining the data from the three transcriptomics datasets (HPA, GTEx, and FANTOM5) using an internal normalization.

Human MEX-3 family members are differentially expressed in healthy tissues of distinct origins, which is summarized in Figure 1. The tissues exerting high MEX-3 protein levels are the male testis (MEX-3B) and the placenta (MEX-3D), which represent immune-privileged tissues avoiding inflammatory reactions through downregulation of classical human leukocyte antigen (HLA) class Ia molecules and upregulation of anti-inflammatory molecules like IL-10 and TGF-β, as well as non-classical HLA class Ib molecules, like HLA-G and HLA-E, respectively. These immune-privileged tissues include the male testis, but also the placenta protecting the developing fetus with its paternal antigens from the maternal immune system, also in the brain and the cornea (Figure 1A) (originally defined as tissues in which foreign tissue grafts can survive for an extended time) [18,19]. Unfortunately, no expression data for the MEX-3A protein are currently available.

MEX-3A, MEX-3B, and MEX-3C mRNA expression was found in the fetal brain, thymus, and testis and in general high MEX-3D mRNA levels were reported in a study also investigating a limited number of other human tissue specimens [8]. There exists conflicting data using TCGA data (Figure 1B), in particular regarding MEX-3D, which is expressed at low levels in lymphoid tissues. In contrast to the MEX-3D mRNA, data from lymphoid tissues were derived from the total RNA panel (BD Biosciences), reported by Buchet-Poyau et al. 2007 [8]. The other MEX-3 proteins are heterogeneously expressed in different tissues. This might reflect their distinct physiological function, which appears to be comparable to that of MEX-3 of *C. elegans*. In addition to testis and placenta, the immune-privileged cerebrum, cerebellum, and ovaries also exhibit higher levels of MEX-3 mRNA. Furthermore, the MEX-3C mRNA can be detected at high levels in primary and secondary lymphoid tissues, in particular in the bone marrow, spleen, and lymph nodes (Figure 1B). Very high levels of MEX-3B, -3C, and -3D mRNA were found in normal testicular tissues.

It is noteworthy that the expression levels of MEX-3 mRNA and proteins do not correlate in all tissues. Transcripts can often be detected with higher frequencies than the corresponding proteins, suggesting that the MEX-3 gene products underlay post-transcriptional or post-translational gene regulation. Such post-transcriptional control might include the regulation by microRNAs (miRs). Indeed, MEX-3C mRNA is targeted by miR-451a [23], although other RBPs might contribute to the stability of the MEX-3 mRNAs. It is even possible that the MEX-3 proteins regulate the mRNA and protein stability of themselves through ubiquitination.

MEX-3 family members predominantly accumulate in the cytoplasm, but can shuttle between the cytoplasm and nucleus. The main molecular characteristics and function of the MEX-3 proteins are summarized in Table 1.

Actual studies report a contribution of human MEX-3 proteins in the regulation of mRNA decay and post-transcriptional control of target genes involved in different physiological processes, including hypertension [43], post-natal growth [44], as well as tumor biology relevant processes, like energy metabolism, apoptosis, and immune responses [9,12,17]. For example, MEX-3C (also known as RKHD2) is an E3 ubiquitin ligase, which plays a role in apoptosis, translational repression, chromosomal instability, energy, homeostasis, obesity, and post-natal growth [17,40,44,45,46,47]. MEX-3D exerts an inverse expression with the anti-apoptotic BCL2 protein in induced-neuronal cells derived from human fibroblasts through an undefined mechanism [48]. In addition, MEX-3A and -B act as components of RNA granules called P bodies [49] and are associated with Argonaute (AGO) proteins as key components of the RNA-induced silencing complex and were demonstrated to be involved in the development of tumors.

## 4. MEX-3 and Tumorigenesis

Recently, RBPs are of growing interest for their ability (i) to play a role in neoplastic transformation and oncogenesis, thereby, affecting the stabilization of tumor-relevant target mRNAs or (ii) to regulate transcription factors that control these tumor-relevant genes. In addition, RBPs might be involved in alternative splicing processes, which are also altered upon malignant transformation [50].

**Figure 2:**
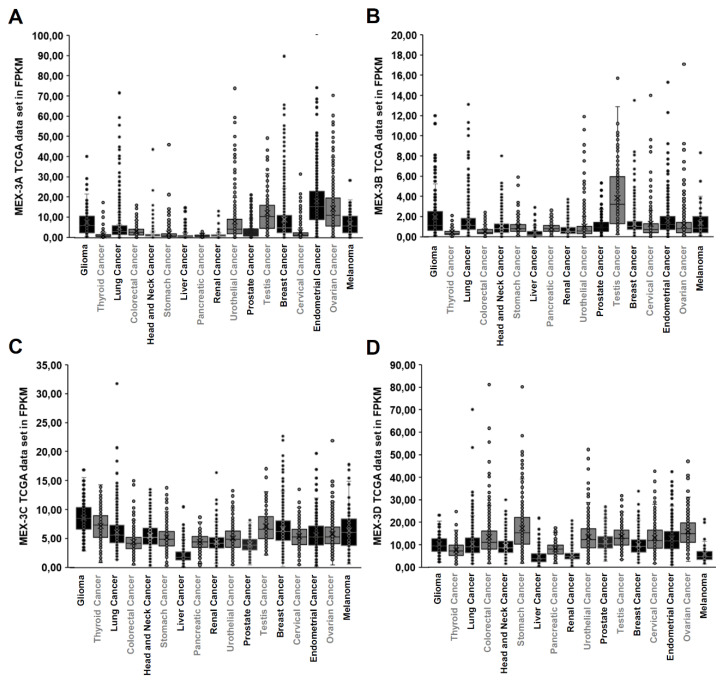
TCGA data sets for MEX-3A-D gene expression in selected human cancers. (**A**–**D**): TCGA expression data for MEX-3A-D in 17 different tumor entities expressed as median FPKM (number fragments per kilobase of exon per million reads) generated by The Human Protein Atlas [20,21,22].

The role of human MEX-3 proteins in tumorigenesis still needs to be defined in much greater detail. In many tumor tissues, MEX-3 homologs are overexpressed when compared to corresponding normal tissues, with some exceptions. As shown in Figure 2, the MEX-3 family members are heterogeneously expressed in the various tumors types, as visualized by the human protein atlas [20,21,22]. While MEX-3A-C showed high expression levels in testicular, endometrial, breast, ovarian, and brain cancer, MEX-3D showed no tumor-type specific increased expression levels. In addition to such TCGA data, a study reported higher expression levels of MEX-3A mRNA levels in human gastric cancer tissues, compared to matched, adjacent, non-cancer tissues, suggesting that human MEX-3A is involved in the development and metastasis formation of this disease [25]. Furthermore, increased MEX-3A levels are also reported in liver cancer, which were significantly associated with a poor patients’ survival [51].

Concerning the functional relevance of MEX-3A expression, its downregulation in gastric cancer cell lines caused a reduced cell proliferation, migration, and transformation capacity. These data suggest that MEX-3A plays a role in the initiation and progression of gastric or liver cancer and might also represent a novel promising therapeutic target for both malignancies [25].

Another study reports that MEX-3C is highly expressed in bladder cancer, but was not detectable in healthy control tissues by immunohistochemistry. This aberrant MEX-3C expression in the bladder cancer, correlated with clinical features, in particular with patients’ prognosis [37]. Furthermore, overexpression of MEX-3C in vitro promotes intracellular adhesion, invasion, and migration. This is associated with an activated JNK signaling by upregulating the downstream protein levels of fatty acid synthase (FASN), the acetyl-CoA-carboxylase-1 (ACC1), and the sterile regulatory element binding protein-1 (SREBP1) [37]. MEX-3C mRNA is targeted by the tumor suppressive miR-451a, which inhibits metastasis formation, as well as proliferation by regulating the epithelial mesenchymal transition (EMT) [52].

The E3 ligase MEX-3B was shown to be involved in the ubiquitination of the runt-related transcription factor 3 (RUNX3), leading to its degradation in gastric cancers [34]. MEX-3B is an important cellular target for the design of epigenetic therapies for chronic hepatitis B virus (HBV) infection, and is associated with a poor prognosis of HBV-associated liver cancer [53]. In addition, MEX-3B could modulate stress-induced apoptosis through post-transcriptional gene regulation of the pro-apoptotic BIM gene, which is induced by MYC and leads to apoptosis by activation of BAX/BAK. This is mediated by the binding of MEX-3B to the 3′ untranslated region (UTR) of BIM, which impairs the interaction with the Ago-miR-92a complex [30,54].

Regarding the underlying mechanisms, through which the MEX-3 proteins are able to induce or contribute to malignant transformation, MEX-3 proteins were shown to ubiquitinate target mRNAs and proteins, marking them to decay. For example, MEX-3A and MEX-3C ubiquitinate the tumor suppressor RIG-I, which affects tumorigenesis in glioblastoma [10,11].

**Figure 3:**
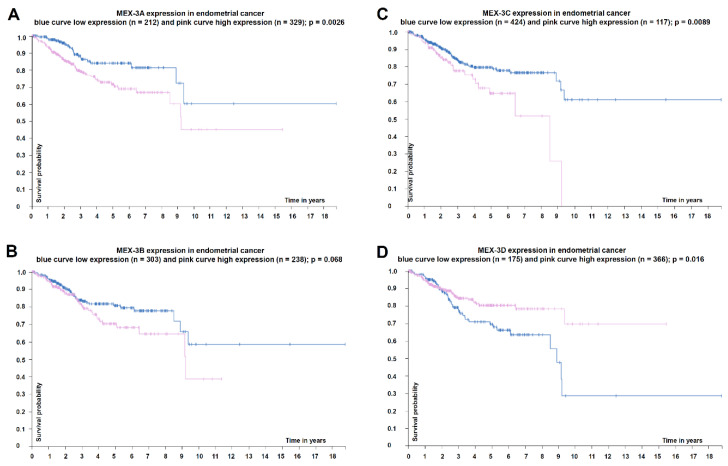
Correlation of the overall survival of endometrial cancer patients’ with MEX-3A–D expression. (**A**–**D**): Kaplan Meier curves for the overall survival of cancer endometrial patients were generated and correlated to the MEX-3A-D expression levels using The Human Protein Atlas [20,21,22].

Furthermore, the expression of the MEX-3 family members was associated with disease progression and patients’ overall survival (OS), but was dependent on the analyzed tumor types (Table 2). Bioinformatics analysis using the Tumor Cancer Genome Atlas (TCGA) revealed low MEX-3A (*p* = 0.0026) and low MEX-3C mRNA (*p* = 0.0089) expression levels in endometrial cancer (no data for testicular germ cell tumors available), which were significantly associated with a better overall survival (OS). Low MEX-3B mRNA levels were also associated with a better, but not statistically significant OS (*p* = 0.068). In contrast, low MEX-3D mRNA levels were associated with a poorer OS (*p* = 0.016) (Figure 3). Despite some exceptions, these tendencies were also detectable in other tumor entities.

It is noteworthy that the observed TCGA data are based on mRNA expression levels, which under consideration of a putative post-transcriptional or post-translational gene regulation might not reflect the in vivo protein-based results.

## 5. MEX-3 and Immune Responses

The classical HLA-Ia expression is essential for cell survival, immune response, and other physiological activities. Foreign antigens, such as viral and tumor antigens presented by HLA-Ia molecules on the cell surface could be recognized by CD8^+^ cytotoxic T lymphocytes (CTL) leading to the elimination of tumor or virus-infected cells. Downregulation, total, or allele-specific loss of HLA-I are a common features of tumors (analogue also in immune privileged tissues) and facilitate CTL-mediated escape [55]. Recently, a post-transcriptional modification of HLA-I mRNAs through ubiquitination was demonstrated, which provides a potential mechanism for controlling the HLA-I turnover, thereby, interfering with immune responses. Some ubiquitin E3 ligases were shown to encode RNA-binding domains (RBD) and were predicted to bind and regulate mRNA [9]. This mechanism could also control the HLA-I antigens in the endoplasmic reticulum (ER) and at the cell surface, suggesting that HLA-I ubiquitination is involved in the post-translational control of the HLA-I assembly.

MEX-3C was shown to be involved in protein regulation/degradation and ubiquitination. Regarding its expression, MEX-3C could be detected in innate immune cells, in particular in activated NK cells, affecting their cytotoxic activity. Recently, a functional siRNA screen identified MEX-3C as a novel RNA-binding E3 ubiquitin ligase, which is responsible for the post-transcriptional regulation of common HLA-A allotypes, without affecting HLA-B and HLA-C [56]. It is able to bind and induce the degradation of HLA-A2 mRNA by binding to its 3′ UTR. The RING domain of MEX-3C is not required for the downregulation of HLA-A2 surface levels, but for the mRNA degradation of HLA-A2, thereby, suggesting a novel mechanism of HLA-I allotype-specific regulation. These results demonstrate a link between ubiquitination and mRNA decay [9]. Ubiquitin is known to regulate the HLA-I mRNA deadenylation, which is required for HLA-I mRNA degradation. It is noteworthy that neither proteasome nor lysosome inhibitors are able to rescue the MEX-3C-mediated HLA-I mRNA degradation, suggesting a non-proteolytic function for ubiquitin in the regulation of the mRNA decay [12]. In addition, MEX-3B could destabilize its own mRNA by binding to the 3′ UTR, which contains elements for mRNA destabilization and translational enhancement.

The function of MEX-3B in immune responses has not yet been well studied, although some analyses demonstrated that MEX-3B can act as a co-receptor of the toll-like receptor 3 involved in the innate anti-viral response [57]. In contrast, the role of MEX-3B in the T cell-mediated anti-tumoral immune responses is not yet analyzed in detail. Interestingly, overexpression of MEX-3B in melanoma cells can downregulate the expression of HLA-A2 by binding to the 3′ UTR of HLA-A, thereby, blocking the recognition and CD8^+^ CTL-mediated killing of tumor cells. This was associated with resistance to immunotherapies [35]. Using melanoma as a model, lower expression of MEX-3B correlated with response to checkpoint blockade, with an antibody directed against the programed cell death 1 (PD-1) receptor, while MEX-3B overexpression inhibited T cell-mediated tumor elimination. These data further suggest that MEX-3 regulates antigen presentation downstream of the interferon (IFN) signaling pathway.

This hypothesis was confirmed by the evaluation of The Cancer Genome Atlas (TCGA) data derived from 150 human testicular germ line tumors, as summarized in Table 3. The expression levels of the *MEX*-*3A*-*D* genes were correlated to the gene expression pattern of selected genes involved in antigen presentation and processing, immune evasion, and IFN-γ signaling. Interestingly, the expression of the *MEX*-*3A*-*D* genes, in particular of *MEX*-*3D*, showed a statistically significant inverse correlation to the expression of antigen presentation and processing components. Furthermore, *MEX*-*3* genes were inversely expressed, compared to the two chaperones calnexin and calreticulin, which are important for the assembly of the HLA-I complex within the ER [58]. This was in contrast to the positive trend of correlated expression of the *MEX*-*3* genes and the components of the IFN-γ signaling pathway.

In the literature, it was already reported that MEX-3B expression leads to a downregulation of HLA-A, thereby inhibiting cancer immunotherapy of anti-PD-1-treated melanoma patients [35]. In melanoma, different immune evasion strategies were reported, which under physiological conditions contribute to the functional immune-privileged tissue microenvironment, including the downregulation of HLA-Ia surface levels, reduced expression of antigen processing machinery (APM) components, as well as induced expression or secretion of immunomodulatory ligands like HLA-G, HLA-E, PD-L1, TGF-β, and IL-10 [58,59,60,61].

The underlying mechanisms of the negative post-transcriptional control of the MEX-3 proteins to HLA molecules or APM components need to be studied in more detail. First evidences demonstrated a direct inhibitory interaction of the MEX-3B protein to the 3′ UTR of the HLA-A mRNA [35]. A similar mechanism was described for MEX3-C downregulating the HLA-A2 mRNA by binding to its 3′ UTR and mRNA ubiquitination [9]. Such interactions might also occur with other MEX-3 proteins and the mRNAs of the APM components, which were statistically significantly inversely correlated, as demonstrated in Table 3.

**Figure 4:**
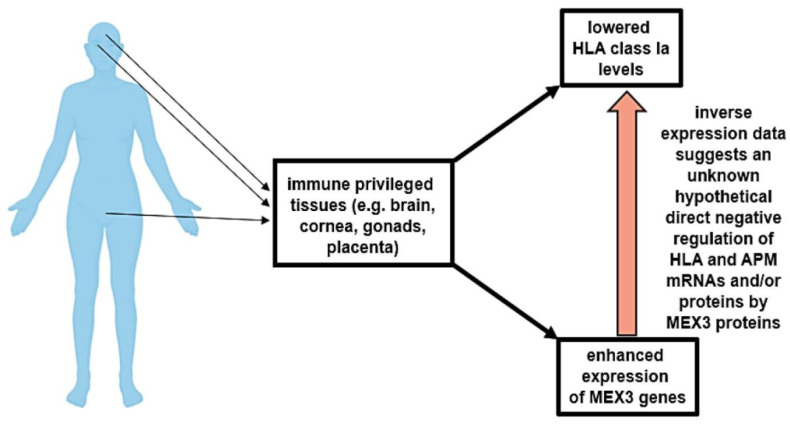
Postulated role of MEX-3 genes in the regulation of HLA class I expression.

It was further hypothesized by the authors that these cellular mechanisms, in combination with already known processes like the promoter methylation of HLA genes [62] and altered expression pattern of regulatory microRNAs [63], might contribute to the physiological low surface levels of HLA class Ia molecules in immune-privileged tissues, as well as to pathophysiological levels in solid and hematopoietic malignancies. Furthermore, enhanced MEX-3 protein expression levels were reported after malignant transformation including gonadal cancers, suggesting that MEX-3 contributes to the tumor immune evasion, by directly targeting HLA-I and APM component encoding gene products, as summarized in Figure 4. To sum up, MEX-3 family members are involved in tumorigenesis and affect immune responses, suggesting their use as potential targets for immunotherapy.

To identify more MEX-3 targeted mRNAs, which might get ubiquitinated or degraded—an immunoprecipiation using MEX-3 specific antibodies in combination with a prior cross-linking could be performed. Furthermore, other issues of MEX-3 activity and function should be addressed. Do MEX-3 proteins exert different affinities for target proteins and for target mRNAs as an acceptor of ubiquitination? Are such differences equal for all of the four MEX-3 proteins? Is mRNA ubiquitination limited to the 3′ UTR of target mRNAs? Are other RNA species (coding or non-coding, mature, or precursors) also ubiquitinated and what would be the effect on cancer biology? Which other factors are involved in the so far not well-understood mechanisms of mRNA ubiquitination and decay?

## 6. Concluding Remarks

The ability of the MEX-3 proteins to negatively regulate the expression of tumor suppressor genes and their reported overexpression, after malignant transformation in several cases, highlights them as putative targets. Whether such intracellular MEX-3 proteins can be targeted by inhibitors or whether putative correlated cell surface markers could be identified by offering the opportunity for immunological therapies should be investigated in more detail.

## Figures and Tables

**Table 1:** Main molecular and cellular characteristics of the MEX-3 homologs in the context of cancer.

MEX-3 Homolog	Main Characterization	Reference
MEX-3A	enhanced cell proliferation and inhibition of apoptosis in bladder cancer	[24]
	enhanced cell proliferation, anchorage-independent growth and migration in gastric cancer	[25]
	higher expression in papillary type bladder urothelial cancer, but no effect on prognosis	[26]
	presence of a nuclear export sequence, connected to colorectal cancer	[27]
	stemness-related gene, upregulation by calcitriol in tumor organoids	[28]
	regulator of post-transcriptional and post-translational control by ubiquitination of target mRNAs/proteins like CDX2 mRNA and the RIG-I protein	[10,29]
MEX-3B	induction of apoptosis by miR-92a targeting Bim	[30]
	inhibition miR-487b-3p and upregulation of IL-33	[31]
	upregulation of CXCL2, induction of neutrophil chemotaxis and migration	[32]
	regulation of Rap1 pathway	[33]
	ubiquitination of Runx3 and increase invasion of gastric cancer cells	[34]
	downregulation of HLA-A expression	[35]
	post-transcriptional regulator of HLA-A	[36]
MEX-3C	suppression of cancer chromosomal instability	[17]
	targeted by miR-451a in colorectal cancer after radio therapy	[23]
	regulation of lipid metabolism through JNK pathway in bladder cancer	[37]
	downregulation in pregnancy-associated breast cancer	[38]
	degradation of MHC I mRNA by ubiquitination	[9]
	activation of NK cells increases MEX-3C levels	[39]
	binding to the MRE10 motif CAGAGUUUAG	[40]
	regulator of post-transcriptional and post-translational control by ubiquitination of target mRNAs/proteins like MHC class I mRNA and the RIG-I protein	[11,12]
MEX-3D	modulation by chemotherapy in AML	[41]
	overexpression in androgen-independent prostate cancer	[42]

**Table 2:** Influence of MEX-3 homologs on the overall survival (OS) of cancer patients.

Cancer	MEX-3A	MEX-3B	MEX-3C	MEX-3D
Bladder	0.333	0.858	0.667	0.016 high better
Breast	0.852	0.399	0.305	0.719
Cervical	0.920	0.839	0.065 low better	0.358
Head and Neck	0.336	0.907	0.757	0.416
Kidney Clear Cell	7.9 × 10^−03^ low better	4.1 × 10^−04^ low better	0.565	0.141
Kidney Papillary	0.415	0.037 low better	0.081 low better	0.036 low better
Liver	5.0 × 10^−03^ high better	0.626	0.017 low better	0.831
Lung	0.072 high better	0.943	0.177	0.033 low better
Skin	0.357	0.139	0.018 high better	0.283

Blue numbers indicate a better patients’ OS with higher expression levels of the MEX-3 homologs in the indicated tumor entity, while the red highlighted numbers represent the correlation of a better OS with lower MEX-3 gene expression levels. Survival data were analyzed with the R2 database (https://hgserver1.amc.nl/), using the appropriate TCGA datasets. The numbers reflect the statistical significance between the OS and the expression levels of the MEX-3 genes with a *p*-value of < 0.1 used as the significance threshold.

**Table 3:** Correlation of the MEX-3A-D expression to the expression of selected immune modulatory genes.

Correlated Expression	MEX-3A	MEX-3B	MEX-3C	MEX-3D
HLA-A	*R* = −0.257 *p* = 1.48 × 10^−03^	*R* = −0.111 *p* = 0.176	*R* = −0.294 *p* = 2.59 × 10^−04^	*R* = −0.447 *p* = 9.97 × 10^−09^
HLA-B	*R* = −0.175 *p* = 0.032	*R* = −0.045 *p* = 0.583	*R* = −0.174 *p* = 0.034	*R* = −0.372 *p* = 2.77 × 10^−06^
HLA-C	*R* = −0.284 *p* = 4.32 × 10^−04^	*R* = −0.126 *p* = 0.125	*R* = −0.254 *p* = 1.70 × 10^−03^	*R* = −0.288 *p* = 3.60 × 10^−04^
B2M	*R* = −0.135 *p* = 0.099	*R* = 0.061 *p* = 0.456	*R* = −0.102 *p* = 0.213	*R* = −0.385 *p* = 1.17 × 10^−06^
TAP1	*R* = −0.161 *p* = 0.050	*R* = 0.019 *p* = 0.819	*R* = −0.039 *p* = 0.635	*R* = −0.376 *p* = 2.14 × 10^−06^
TAP2	*R* = −0.167 *p* = 0.041	*R* = 0.046 *p* = 0.579	*R* = −0.013 *p* = 0.877	*R* = −0.339 *p* = 2.15 × 10^−05^
TPN	*R* = −0.134 *p* = 0.103	*R* = −0.032 *p* = 0.699	*R* = −0.293 *p* = 2.76 × 10^−04^	*R* = −0.457 *p* = 4.02 × 10^−09^
CALR	*R* = −0.329 *p* = 3.95 × 10^−05^	*R* = −0.520 *p* = 8.70 × 10^−12^	*R* = −0.458 *p* = 3.67 × 10^−09^	*R* = 0.075 *p* = 0.362
CANX	*R* = −0.526 *p* = 4.65 × 10^−12^	*R* = −0.483 *p* = 3.99 × 10^−10^	*R* = −0.133 *p* = 0.105	*R* = −0.182 *p* = 0.026
ERP57	*R* = −0.069 *p* = 0.404	*R* = −0.036 *p* = 0.661	*R* = 0.015 *p* = 0.858	*R* = −0.155 *p* = 0.058
ERAP1	*R* = −0.114 *p* = 0.165	*R* = 0.034 *p* = 0.681	*R* = −0.132 *p* = 0.107	*R* = −0.133 *p* = 0.104
ERAP2	*R* = −0.010 *p* = 0.907	*R* = −0.023 *p* = 0.779	*R* = −0.126 *p* =0.123	*R* = −0.295 *p* = 2.52 × 10^−04^
LMP2	*R* = −0.150 *p* = 0.068	*R* = −0.036 *p* = 0.661	*R* = −0.159 *p* = 0.053	*R* = −0.412 *p* = 1.68 × 10^−07^
LMP7	*R* = −0.162 *p* = 0.048	*R* = −0.022 *p* = 0.788	*R* = −0.255 *p* = 1.64 × 10^−03^	*R* =−0.398 *p* = 4.55 × 10^−07^
LMP10	*R* = −0.201 *p* = 0.014	*R* = −0.161 *p* = 0.049	*R* = −0.340 *p* = 2.02 × 10^−05^	*R* =−0.521 *p* = 8.59 × 10^−12^
PDL1	*R* = −0.077 *p* = 0.347	*R* = 0.060 *p* = 0.469	*R* = 0.302 *p* = 1.72 × 10^−04^	*R* = −0.301 *p* = 1.80 × 10^−04^
HLA-E	*R* = −0.236 *p* = 3.61 × 10^−03^	*R* = −0.004 *p* = 0.963	*R* = −0.089 *p* = 0.280	*R* = −0.329 *p* = 3.94 × 10^−05^
HLA-G	*R* = −0.410 *p* = 1.89 × 10^−07^	*R* = −0.176 *p* = 0.032	*R* = −0.168 *p* = 0.040	*R* = −0.210 *p* = 9.92 × 10^−03^
IFNG	*R* = −0.087 *p* = 0.291	*R* = 0.015 *p* = 0.859	*R* = 0.160 *p* = 0.050	*R* =−0.249 *p* = 2.08 × 10^−03^
IFNGR1	*R* = −0.035 *p* = 0.674	*R* = 0.135 *p* = 0.099	*R* = −0.038 *p* = 0.641	*R* = −0.478 *p* = 6.38 × 10^−10^
IFNGR2	*R* = 0.054 *p* = 0.515	*R* = −0.054 *p* = 0.514	*R* = −0.430 *p* = 3.95 × 10^−08^	*R* = −0.178 *p* = 0.029
JAK1	*R* = 0.204 *p* = 0.012	*R* = 0.517 *p* = 1.24 × 10^−11^	*R* = 0.288 *p* = 3.46 × 10^−04^	*R* = −0.138 *p* =0.093
JAK2	*R* = 0.145 *p* = 0.076	*R* = 0.398 *p* = 4.56 × 10^−07^	*R* = 0.266 *p* = 9.85 × 10^−04^	*R* = −0.296 *p* = 2.31 × 10^−04^
STAT1	*R* = 0.116 *p* = 0.158	*R* = 0.219 *p* = 7.00 × 10^−03^	*R* = 0.266 *p* = 1.01 × 10^−03^	*R* = −0.204 *p* = 0.012
IRF1	*R* = −0.171 *p* = 0.036	*R* = 0.046 *p* = 0.580	*R* = −0.047 *p* = 0.566	*R* = −0.438 *p* = 2.13 × 10^−08^

Based on TCGA data sets of 150 testicular germ cell tumors, the expression data of the MEX-3 mRNAs were correlated to the expression of HLA-Ia und –Ib, APM components, PDL1, and the molecules involved in the IFN-γ signaling pathway (http://r2.amc.nl; transform_2log, plotted into Y-Y-plot; p value is significance of correlation). Statistically significant inverse correlated genes are highlighted in red, statistically significant positive correlated genes in blue. (For a better estimation—the very strong correlation of IFN-γ and one of its directly induced target genes ICAM1 is *R* = 0.601 and *p* = 4.07 × 10^−16^.).

## Abbreviations

ACC1	acetyl-CoA-carboxylase-1
APM	antigen processing machinery
*C. elegans*	*Caenorhabditis elegans*
CTL	cytotoxic T lymphocyte
EMT	epithelial mesenchymal transition
ER	endoplasmic reticulum
FASN	fatty acid synthase
HBV	hepatitis B virus
HLA	human leukocyte antigen
hnRNP	heterogeneous nuclear ribonucleoprotein
HOTAIR	HOX antisense intergenic RNA
IFN	interferon
KH	K-homology
MEX-3	mex-3 RNA-binding family member
MHC	major histocompatibility complex
MHC-I	MHC class I
miRNA	microRNA
OS	overall survival
PD-1	programed death receptor 1
RBD	RNA-binding domain
RBP	RNA-binding protein
RUNX3	runt-related transcription factor 3
SREBP1	sterol regulatory element-binding protein 1
TCGA	The Tumor Cancer Genome Atlas
UTR	untranslated region
